# New Bird Sexing Strategy Developed in the Order Psittaciformes Involves Multiple Markers to Avoid Sex Misidentification: Debunked Myth of the Universal DNA Marker

**DOI:** 10.3390/genes12060878

**Published:** 2021-06-07

**Authors:** Aleksandra Kroczak, Magdalena Wołoszyńska, Heliodor Wierzbicki, Marcin Kurkowski, Krzysztof Aleksander Grabowski, Tomasz Piasecki, Livio Galosi, Adam Dawid Urantówka

**Affiliations:** 1 Department of Genetics, Wrocław University of Environmental and Life Sciences, Kożuchowska 7, 51-631 Wrocław, Poland; aleksandra.kroczak@upwr.edu.pl (A.K.); magdalena.woloszynska@upwr.edu.pl (M.W.); heliodor.wierzbicki@upwr.edu.pl (H.W.); marcin.kurkowski94@gmail.com (M.K.); chudy.kg@gmail.com (K.A.G.); 2 Department of Epizootiology and Clinic of Bird and Exotic Animals, Wrocław University of Environmental and Life Sciences, pl. Grunwaldzki 4, 550-366 Wroclaw, Poland; tomasz.piasecki@upwr.edu.pl; 3 School of Biosciences and Veterinary Medicine, University of Camerino, Via Circovallazione 93/95, 62-024 Matelica, Italy; livio.galosi@unicam.it

**Keywords:** chromosomes Z/W, gene CHD1, gene NIPBL, molecular markers, Psittaciformes, sex determination

## Abstract

Sexing of birds is indispensable for scientific, breeding and conservation programs but is difficult in many species and is particularly problematic in the case of nestlings showing no sexual dimorphism. Most useful and efficient methods of sex determination are based on unique features of the Z and W sex chromosomes detected via PCR to distinguish males (ZZ) and females (ZW). During the last twenty-five years researchers searched for the universal marker capable of sexing a maximally wide spectrum of species in a single PCR assay. We screened the phylogenetically representative set of 135 Psittaciformes species including 59 species sexed for the first time. Two known (P2P8, CHD1iA) PCR markers and four additional W/Z polymorphisms (CHD1iE, CHD1i16, CHD1i9 and NIPBLi16) located within the Chromo Helicase DNA binding CHD1 or the Nipped-B homolog NIPBL genes were applied. We present the electrophoretic patterns obtained for the PCR products of the analyzed markers including most typical and atypical patterns allowing sex determination, as well as those obtained when the given marker failed in sexing. Technical aspects of molecular sex determination are discussed: the optimization of amplification conditions, direct PCR and potential misinterpretations. A truly universal marker has not been found, and therefore, we propose a sexing strategy based on multiple CHD1i16, NIPBLi16, CHD1i9 and CHD1iE markers. This new strategy confirms the sex of a given bird with at least two markers detecting independent Z/W polymorphisms, reduces the number of necessary PCR reactions and minimizes the risk of sex misidentification.

## 1. Introduction

Psittaciformes (parrots) is the fourth largest bird order (after Passeriformes, Caprimulgiformes and Piciformes) and is presently considered to be the most threatened because as much as 28% of extant species are classified as threatened under IUCN criteria [1]. According to the Red List Index, parrots have the highest extinction risk compared to other avian orders. Sadly, the extinction risk will likely rise, as many parrot species are large-bodied and slow-breeding, and most taxa are ecologically specialized, which is known to be associated with extinction [1]. Parrots have always been appreciated as exotic and ornamental animals. However, over the years, parrots have become real pets due to their beautiful plumage, high intelligence, strong and charming personalities and social behavior. As a consequence, parrots have become the most captivating avian group [2]. Increased demand for captive-bred companion parrots increases their illegal trapping from the wild, which additionally strongly influences their extinction risk, along with such threats as habitat loss or degradation, a biased sex ratio in adults and low female breeding participation (reviewed by Heinsohn et al. [3]). Hence, the breeding, monitoring and conservation of endangered parrot populations are particularly important and require an efficient sexing method.

Accurate sex typing is a serious pitfall in many scientific, breeding and conservation programs, as in over 50% of the world bird species, nestlings, as well as adult individuals, show no sexual dimorphism. Identification of an efficient and universal method of sex determination is of particular importance in the case of the Psittaciformes, as most parrot species show no sexual dimorphism. Therefore, parrots represent the right avian model for such studies, especially because this group is strongly diversified phylogenetically.

The unequivocal identification of the sex of individuals appears essential in conservation and reintroduction projects. However, when selecting founders, sex may be uncertain for nestlings of most species [4]. As a result this may lead to inefficient results or negative impacts on conservation efforts. The proportion of males and females is an important factor for success in a newly founded population. The sex ratio among sexually mature individuals is often male-biased for monogamous species [5]. Random fluctuations of sex ratio in a small, protected population could lead to increased extinction probability as well as affect effective population size [4]. Therefore, accurate sex determination is crucial for assessing the growth and viability of endangered bird populations.

The best known sexing method for birds is genetic typing based on DNA features unique to each gender. Molecular tests, specifically those exploiting PCR technology, are reliable, simple, cheap, fast and have the advantage that DNA can be extracted from easily available samples like feathers.

Birds are female heterogametic organisms (females ZW and males ZZ), suggesting that chromosome W, present only in females, should be a perfect source of sequences useful as DNA markers for sex-typing. However, like chromosomes Y in male heterogametic organisms, the avian W chromosomes, are in most cases, highly heterochromatic and degenerated variants of once-recombining proto-sex chromosomes [6,7] and contain large amounts of repetitive sequences [8]. Such sequences poorly perform in all types of DNA analyses and evolve rapidly, even between closely related species; therefore, they provide sex-linked markers of a limited range [9]. To obtain a universal marker for the successful molecular sexing of a maximally wide spectrum of species, genes were explored as potential markers since coding regions are better conserved among the species. Particularly useful are genes with a gametologous copy on the Z chromosome, since markers targeting both W and Z copies of a gene and detecting polymorphism between the two copies allow for sex determination but also eliminate the false negative detection of males that may occur when only the W chromosome is targeted by a marker. In the case of the PCR markers, two products are amplified on female DNA because Z and W copies of the gene show length polymorphism, and a single product appears in the male sample since gene copies from two Z chromosomes are usually identical in length. Primers are designed to anneal to conserved sequences of neighbor exons flanking Z/W polymorphic introns. Sequences of more than 40 genes from the avian W chromosome are known to have a gametologous Z copy [10]. However, for historical reasons, the most widely used PCR markers for sex-typing in birds is based on the first discovered avian W chromosome gene—Chromo Helicase DNA binding gene (CHD1) [11,12]. Other genes having different forms in the W and Z chromosomes were also shown as potential sex differentiation markers: the spindlin SPIN gene [13], the Nipped-B homolog gene (NIPBL) [14] and the RAS p21 protein activator 1 gene (RASA1) [15].

Here we applied two well-known and widely used PCR markers (P2P8, [16]; CHD1iA, [12]) and four other W/Z length polymorphisms (CHD1iE, CHD1i16, CHD1i9 and NIPBLi16) located within CHD1 and NIPBL genes to analyze their performance in sex determination on male/female pairs of a phylogenetically representative group of 135 Psittaciformes species. The majority of these species (76) were already sexed [11,13,16,17,18,19,20,21,22,23,24,25,26,27,28,29,30,31,32,33], but 59 taxons were sexed for the first time in our study. Our aims were (1) to find a marker that would combine the advantages of being universal (able to successfully sex as many species as possible) and easy to use and (2) to provide the guidelines for optimizing PCR conditions during the amplification of the markers using DNA templates isolated from fresh blood, feathers and more challenging dried blood samples.

## 2. Materials and Methods

### 2.1. DNA Extraction and Biological Samples

We analyzed blood or feather samples from 135 parrot species listed in Table 1. Blood samples were taken as dry blood spots on a fiber filter for laboratory analysis (no fresh blood was used in this study). Collecting blood on such material allows the blood to soak evenly, preventing the formation of blood clots on the filter surface, which could cause the inefficient washing out of blood cells and their ineffective digestion with proteinase K in the first stage of DNA isolation. Drying the blood on a filter is considered a good preservative method because it does not contain water due to evaporation, thus stopping the potential process of microbial breakdown of blood cells and genomic DNA degradation. Such preparation of samples enables their safe transport by courier over long distances. The collected blood samples were preserved in parafilm-sealed Eppendorf tubes at −20 °C until use to avoid dampness. In the case of freshly collected breast feather samples, feather shafts were cut off and stored in the same conditions until use. Total DNA was extracted from both tissue types with Sherlock AX Kit (A&A Biotechnology, Gdynia, Poland) according to the manufacturer’s protocol.

Blood samples of *Nestor notabilis, Coracopsis vasa, Primolius couloni and Anodorhynchus hyacinthinus* individuals were obtained from Zoological Garden in Wroclaw, Poland and Zoological Garden in Warsaw, Poland, respectively. They were collected by veterinarians while administering standard veterinary care. Blood samples of *Cyanopsitta spixii, Amazona versicolor and Amazona guildingii* individuals were obtained from ACTP (Association for the Conservation of Threatened Parrots e.V.) with appropriate CITES permissions: DE-LB-160216-0075, DE-LB-160216-0076, DE-LB-160216-0077, DE-LB-160420-0342, DE-LB-160420-0343, DE-LB-160420-0344, DE-LB-160420-0345, DE-LB-160420-0346, DE-LB-160420-0347. Feather samples of *Calyptorhynchus banksii* individuals were obtained from Rosewood Bird Gardens & Breeding Farms (in Rosewood, Australia) with CITES permission no.: PWS2017-AU-001349. Feather samples from *Alipiopsitta xanthops*, two *Enicognathus* species and nine *Amazona* taxa (*A. agilis*, *A. brasiliensis*, *A. collaria*, *A. dufresniana*, *A. festiva*, *A. farinose guatemalae*, *A. rhodocorytha*, *A. tucumana*, *A. xantholora*) were obtained from Loro Parque Foundation (Avda Puerto de la Cruz, Tenerife, Spain). Blood samples from *Psittacara brevipes* and *Rhynchopsitta terrisi* individuals were collected by Africam Safari (Boulevard Capitán Carlos Camacho Espíritu Km 16.5, Oasis, 72960 Puebla, Mexico) veterinarians and imported with CITES permissions no.: 12PL000353/MZ. All other samples (blood stains or breast feathers) examined in this study came from birds kept in Polish and Italian private breeding centers and facilities. They were collected by veterinarians during their routine clinical practice as the result of periodic veterinary examinations of birds. 

### 2.2. DNA Amplification

The PCR amplifications were performed in a total 25 μL of the reaction mixture containing 50 ng of the DNA template, 1U DreamTaq Green DNA Polymerase (Thermo Fisher Scientific, Waltham, MA, USA), 2.5 μL of 10 × buffer, 0.6 μL of 10 mM dNTPs, and 0.6 μL of each primer (10 μM). The reaction conditions were as follows: the P2/P8 program: 94 °C 5 min, (94 °C 30 s, 48 °C 30 s, 72 °C 45 s) 35 times, and 72 °C 5 min; the CHD1iA program: 94 °C 5 min, (94 °C 30 s, 50 °C 30 s, 72 °C 90 s) 35 times, and 72 °C 5 min; the CHD1i16 program: 94 °C 5 min, (94 °C 30 s, 53 °C 30 s, 72 °C 90 s) 35 times, and 72 °C 5 min; the CHD1i9 program: 94 °C 5 min, (94 °C 30 s, 51 °C 30 s, 72 °C 60 s) 35 times, and 72 °C 5 min; the NIPBLi16 program: 94 °C 5 min, (94 °C 30 s, 53 °C 30 s, 72 °C 60 s) 35 times, and 72 °C 5 min; the CHD1iE **program 1**: 5 times [94 °C 30 s, 62 °C (touchdown 2°/cycle) 30 s, 72 °C 45 s], 27 times (94 °C 30 s, 50 °C 30 s, 72 °C 45 s), and 72 °C 5 min; the CHD1iE **program 2**: 10 times [94 °C 30 s, 62 °C (touchdown 1°/cycle) 30 s, 72 °C 90 s], 27 times (94 °C 30 s, 42–52 °C 30 s, 72 °C 90 s), and 72 °C 5 min; the CHD1iE **program 3**: 5 times [94 °C 30 s, 62 °C (touchdown 2°/cycle) 30 s, 72 °C 90 s], 27 times (94 °C 30 s, 42–52 °C 30 s, 72 °C 90 s), and 72 °C 5 min; the CHD1iE **program 4**: 94 °C 5 min, (94 °C 30 s, 42–52 °C 30 s, 72 °C 90 s) 32 times, and 72 °C 5 min; the CHD1iE **program 5**: 94 °C 5 min, (94 °C 30 s, 42–52 °C 30 s, 72 °C 30 s) 32 times, and 72 °C 5 min. In the case of direct PCR amplifications, all components and appropriate PCR conditions were maintained, but instead of isolated DNA, the fragment of dry blood spot (area of about one square millimeter) was used as a template.

## 3. Results and Discussion

Accurate and efficient sexing of birds remains challenging for scientists, ecologists, conservationists and breeders due to the lack of sexual dimorphism in many species and all nestlings, as well as unsuccessful attempts to discover universal and easily applied molecular markers. We have addressed this issue in the Psittaciformes order by testing six W/Z polymorphisms, including two well-known markers (P2/P8; CHD1iA). We did not find a universal marker, and we evaluated the potential risks of sex misidentification associated with the application of only one marker. Finally, we developed a strategy of sex identification based on multiple markers. 

### 3.1. The Set of Parrot Species Representative for Phylogenetic Divergence of Psittaciformes Order

Parrots represent one of the most species-rich orders of birds, with 373 species grouped within 89 genera [34]. According to present parrot taxonomy (Table 2), three superfamilies are recognized [35], which are subsequently divided into families, subfamilies, tribes and genera [35,36]. In this study, we examined 135 species belonging to 56 genera (Table 1), which makes our study the most comprehensive so far and truly taxonomically/phylogenetically representative. 

### 3.2. Genomic Localization of the Analyzed Markers

**Figure 1:**
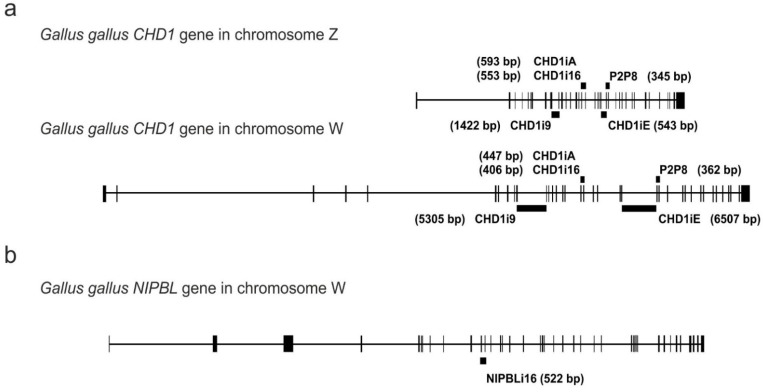
The location of the P2P8, CHD1iA, CHD1i16, CHD1i9, CHD1iE and NIPBLi16 molecular markers within the CHD1 gene gametologous copies in the chromosomes Z and W (**a**), and the NIPBL gene copy located in the chromosome W (**b**) of *Gallus gallus*. The vertical bars of various thicknesses correspond to exons; the horizontal lines between the exons are introns. The thick black lines above or below the introns represent indicated markers as the PCR products covering respective intron and amplified using the primers located within the flanking exons. The sizes of the PCR products are written in brackets.

To illustrate the genomic localization of the analyzed markers, we show their position within the CHD1 or NIPBL genes in the chicken (*Gallus gallus*) sex chromosomes (Figure 1). The CHD1 markers of both W and Z chromosomes are visualized to illustrate sex-specific length polymorphisms (Figure 1a). The position of the NIPBL intron is indicated only in the chromosome W (Figure 1b) because retroposon insertion in chromosome Z resulting in Z/W polymorphism occurs in Neoaves and therefore is not present in chicken [14].

### 3.3. The Male- and Female-Specific Patterns of the P2P8 Marker Are Difficult to Distinguish

The P2P8 marker was described for the first time and successfully applied to 27 out of 28 species sampled across the class Aves by Griffiths et al. [16]. The marker was later shown to sex approximately 80% of non-ratite species [37]. It was named after the P2 and P8 primers used to amplify the entire intron of the CHD1 gene showing length polymorphism between two gametologous W and Z copies. In a majority of tested bird species, the chromosome-W-specific PCR product was larger compared to chromosome Z, although the size difference was small and ranged from 10 to maximally 50 bp depending on the species [16,22]. In chicken, the 23rd intron is amplified with P2 and P8 primers, and the products derived from the chromosomes W and Z are 362 bp and 345 bp and differ by 17 bp due to the insertions of 10 bp and 8 bp and a deletion of 1 bp in the CHD1W copy. To separate the PCR products obtained in female individuals, electrophoresis was performed using 3% [16] or 5% [38] agarose gels or even, for some species, polyacrylamide gels [17].

**Figure 2:**
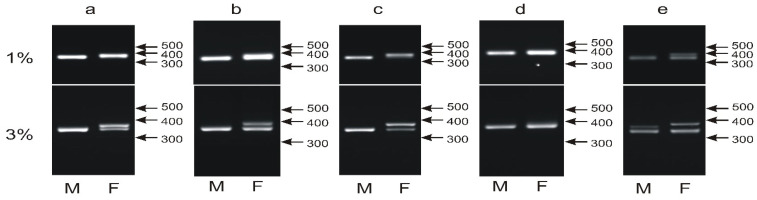
Different patterns of the PCR products representing the P2P8 marker obtained for male (M) and female (F) birds of *Guaruba guarouba* (**a**), *Amazona barbadensis* (**b**), *Neopsephotus bourkii* (**c**), *Cacatua sulphurea* (**d**) and *Neophema pulchella* (**e**) species and resolved in 1% or 3% agarose gel. Arrows and numbers correspond to location and size (in bp) of the DNA molecular marker run on the same gel.

At least 68 parrot species have been sexed using the P2P8 marker [16,17,22,38,39,40], showing a typical pattern of one band in males and two bands of very similar sizes in females. We applied the P2P8 marker to 18 from our selection of 135 species including taxons that have already been sexed with this or another marker or species sexed for the first time in our study. The majority of 16 species produced the typical pattern of PCR products (Figure 2a–c), while for two species, sex determination with the P2P8 marker was unsuccessful. The male and female individuals of *Cacatua sulphurea* showed an identical pattern of a single band indicating either no or very short length polymorphism between CHD1W and CHD1Z within the tested region (Figure 2d). In the case of *Neophema pulchella,* an additional band was visualized in the male sample with a size intermediate between the CHD1Z and CHD1W bands (Figure 2e).

In conclusion, firstly, the P2P8 marker has a serious technical disadvantage since the PCR products derived from the CHD1W and CHD1Z copies are difficult to resolve in agarose gel and secondly, the marker is not universal, since in our relatively small survey of 18 parrot species, two species failed to be sexed. Therefore, we decided to abandon P2P8 in our search for a universal and easily applicable marker.

### 3.4. The CHD1iA Marker Shows Limitations in Sex Determination While Tested in New Species

The CHD1iA marker (CHD1, intron A [23]) is based on the W/Z length polymorphism of the CHD1, intron 17 in the CHD1W and CHD1Z genes of *Gallus gallus* (Figure 1a). The CHD1, intron A was amplified using primers 2550F and 2718R [12] and had sizes of 593 bp (CHD1Z) and 447 bp (CHD1W). The CHD1iA marker has been applied to sex 50 non-ratite bird species from 11 orders through the avian phylogeny and worked successfully for 47 species. The three remaining species were sexed with an alternative pair of primers targeting the same intron [12].

**Figure 3:**
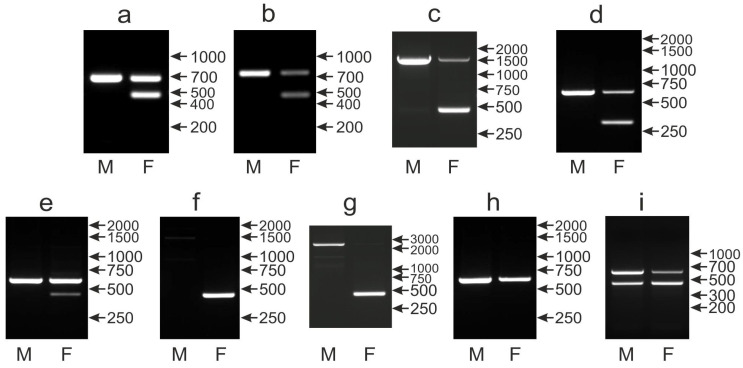
Different patterns of the PCR products representing the CHD1iA marker obtained for male (M) and female (F) birds of *Ara chloroptera* (**a**), *Myiopsitta monachus* (**b**), *Forpus xanthops* (**c**), *Alisterus scapularis* (**d**), *Agapornis nigrigenis* (**e**), *Psittacula eupatria* (**f**), *Psittacula cyanocephala* (**g**), *Platycercus icterotis* (**h**) and *Orthopsittaca manilata* (**i**) species. Arrows and numbers correspond to location and size (in bp) of the DNA molecular marker run on the same gel.

In our survey of 135 parrot species, the CHD1iA marker performed well for 113 species wherein males and females were clearly distinguished. The most typical pattern with the CHD1Z band (650 bp) in males and CHD1Z and CHD1W (450 bp) bands in females was found in 105 species (Figure 3a). In three species, similar patterns were observed but the Z band was larger: about 750 bp in *Myiopsitta monachus* (Figure 3b) or about 1500 bp in *Forpus coelestis* and *Forpus xanthops* (Figure 3c). Finally, in another five species, the W band was slightly smaller (about 300) bp in *Polytelis alexandrae*, *Polytelis swainsonii*, *Alisterus scapularis*, *Aprosmictus erythropterus* and *Polytelis anthopeplus* (Figure 3d).

In 13 out of 22 remaining species, sexing was still possible, although some bands were missing or low in intensity. In five species: *Agapornis canus*, *Agapornis nigrigenis*, *Barnardius zonarius*, *Platycercus elegans* and *Rhynchopsitta terrisi,* a normal pattern of bands was detected, but the CHD1W band in female was very weak (Figure 3e), possibly due to not-full primer homology, which could lead to serious doubts during sex determination. The single CHD1W band in females and no band in males were detected in four species: *Eclectus roratus*, *Psittacula alexandrii*, *P. eupatria* and *P. krameri* (Figure 3f) making sexing possible but without the positive control of the CHD1Z, which may lead to the false determination of males in cases of unsuccessful PCR. In *Psittacula derbiana, Psittacula cyanocephala*, *Psittinus cyanurus* and *Forpus crassirostris,* females had normal band CHD1W, while the CHD1Z band of expected size was not detected, and instead, the high-molecular band was visualized only in males (Figure 3g). In the last two cases the sequence mutation rearrangement of chromosome Z might be responsible for the alternated PCR pattern. 

Sexing was not possible in six species, including *Anodorhynchus hyacinthinus*, *Neophema elegans*, *N. pulchella*, *N. splendida*, *Platycercus caledonius* and *P. icterotis*, because only one band was amplified (Figure 3h), corresponding to CHD1Z while the CHD1W band was absent, apparently due to none or only small differences between intron A of Z and W copies of CHD1. Finally, sexing was also impossible in three other species (*Coracopsis vasa*, *Nestor notabilis* and *Orthopsittaca manilata*), wherein both in males and in females, the two Z and W bands were visible (Figure 3i) in slightly different proportions. 

Taking together, in 113 parrot species, the CHD1iA marker produced patterns of a single Z band in males and two Z and W bands in females allowing simple and unambiguous sexing. In the remaining 22 species, only 9 species presented identical patterns in males and females, and therefore, sex determination was impossible. Consequently, although the CHD1iA marker can be successfully used for a majority of parrots, it is not universal, and therefore, there is a need to search for new markers that are universal, or at least complementary, to CHD1iA.

### 3.5. Application of New CHD1iE Marker Is Difficult Due to Technical Drawbacks in PCR

The length polymorphism of the CHD1W/Z intron E (the 22nd intron of CHD1 in *Gallus gallus*) was described by Sundstrom et al. [23] for 18 avian species, including three parrot taxa: *Aratinga acuticaudata*, *Bolborhynchus lineola* and *Pyrrhura frontalis*. To check whether this polymorphism can be applied as a molecular marker for sex determination, we performed optimization of the PCR conditions for the amplification of the intron with original primers [23]. In chickens, the primers should produce DNA fragments of 543 bp (CHD1Z) and 6507 bp (CHD1W). With the use of *Guaruba guarouba* as a template, we tested five amplification programs, including the original one (Program 1, Supplementary Figure S1a) and four variant programs wherein elongation time was modified (Programs 2, 3, 4, 5) and/or the ramping of the touch down step was changed (Program 2) or the touch down step was omitted (Programs 4, 5). For each variant program, the annealing temperature was optimized in the range 42–52 °C (Supplementary Figure S1b). In all cases, a PCR product of about 1000 bp was obtained only when the female DNA was used as a template and therefore, corresponded to CHD1W. The specific and efficient amplification of CHD1Z fragments of about 3300 bp was dependent on PCR reaction parameters (Supplementary Figure S1b). Finally, Program 2 (elongation time 90 s, ramping of touch down 1 °C/cycle with annealing temperature of 52 °C) yielded most abundant and specific fragments of both CHD1Z and CHD1W (with lengths of 3300 bp and 1000 bp, respectively) and was selected for further analyses. However, in the case of another few tested species, we were not able to amplify the CHD1Z product. Therefore, sexing with the marker CHD1iE was based only on the presence/absence of the chromosome-W-specific band of 1000 bp. Programs 2 and 5 (elongation time 30 s; annealing temperature of 48 °C) were applied to test marker CHD1iE in the sex determination of 64 parrot species (Table 1).

**Figure 4:**
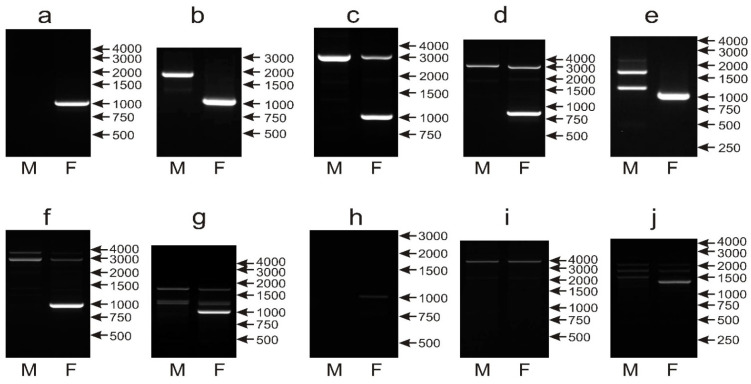
Different patterns of the PCR products representing the CHD1iE markers obtained for male (M) and female (F) birds of *Amazona finschi* (**a**), *Cyanoliseus patagonus* (**b**), *Barnardius zonarius* (**c**), *Agapornis nigrigenis* (**d**), *Psittacula derbiana* (**e**), *Lophochroa leadbeateri* (**f**), *Psittacula eupatria* (**g**), *Trichoglossus haematodus* (**h**), *Alisterus scapularis* (**i**) and *Nestor notabilis* (**j**) species. Arrows and numbers correspond to location and size (in bp) of the DNA molecular marker run on the same gel.

For the majority of the analyzed species, the same patterns were obtained when two programs were applied; only in 11 samples were differences observed and, in these cases, the pattern easier for interpretation was considered (the 1000 bp band present in the female sample, no or fewer unspecific bands). Sex determination with the CHD1iE marker was possible in 54 out of 64 species. In 38 species, the most typical pattern was observed with the CHD1W 1000 bp band detected in females, and no band obtained in the male samples (Figure 4a). In 10 species, in addition to the CHD1W band of 1000 bp, a single PCR product of 2000 bp (*Cacatua sulphurea*, *Cyanoliseus patagonus*) or 3000 bp *(Guaruba guarouba*, *Pionites melanocephalus*, *Psephotus haematonotus*, *Agapornis roseicollis*, *Eolophus roseicapillus*, *Barnardius zonarius*, *Primolius couloni* and *Agapornis nigrigenis*) was detected (Figure 4b–d). In 6 species, the 1000 bp band was amplified in the female sample, and sex determination was possible, but unspecific bands were visible (Figure 4e–g) in male (*Psittacula derbiana*, *Aratinga nenday*, *Anodorhynchus hyacinthinus*) female (*Loriculus galgulus*) or in both samples (*Lophochroa leadbeateri*, *Psittacula eupatria*). Finally, sexing was not possible in 10 species (*Bolborhynchus lineola*, *Forpus crassirostris*, *Polytelis alexandrae*, *Polytelis swainsonii*, *Polytelis anthopeplus*, *Trichoglossus haematodus*, *Alisterus scapularis*, *Aprosmictus erythropterus*, *Chalcopsitta duivenbodei*, *Nestor notabilis*), which yielded extremely weak (Figure 4h) or no 1000 bp band (Figure 4i,j).

Despite PCR optimization efforts, the chromosome Z band of the CHD1iE marker could not be reproducibly amplified, and unspecific or large size bands were frequently obtained. Moreover, the predicted lengths of the chicken intron E amplified with primers used in our study (6507 bp CHD1W and 543 bp CHD1Z) suggest that the optimization of the PCR conditions to amplify intron E might be difficult, not only in parrots but also in other avian taxa due to large and variable sizes of CHD1W/Z copies.

Summing up, we do not consider this marker as a good candidate for routine applications in parrot sexing, although it can be used to resolve difficult cases where other markers fail. For example, the *Myiopsitta monachus* and *Poicephalus gulielmi* species cannot be sexed or give atypical patterns with the CHD1i9 and NIPBLi16 markers, while the CHD1iE (Figure 4a) marker allows the distinguishing of males and females and therefore, can be used together with CHD1iA for sex determination in these species (Table 1).

### 3.6. None of the CHD1i16, CHD1i9 and NIPBLi16 Markers Turn Universal When Applied to the Sexing of a Large Number of Parrot Species

To find a new tool giving homoplasy-free phylogenetic signals and to investigate the evolution of avian sex chromosomes, Suh et al. [14] screened 126 sequenced intron pairs of 12 gametologous genes looking for retroposon insertions. The CR1 (chicken repeat 1 family of long interspersed element) and LTR (long terminal repeat element of endogenous retroviruses) retroposons were found in the neognathous CHD1Z introns 16 and 9 (introns 17 and 10 in Figure 1), respectively, and were absent in the CHD1W gametolog. Another CR1 retroposon was only identified in the Z gametolog of the NIPBL intron 16 in Neoaves and was not detected in the galloanseran lineage. 

**Figure 5:**
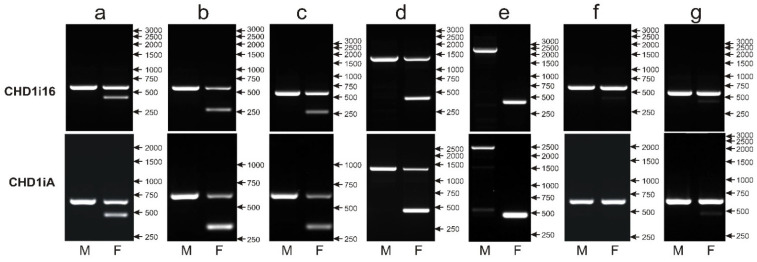
Different patterns of the PCR products representing the CHD1i16 (upper panel) and CHD1iA (lower panel) markers obtained for male (M) and female (F) birds of *Guaruba guarouba* (**a**), *Polytelis alexandrae* (**b**), *Polytelis swainsonii* (**c**), *Forpus crassirostris* (**d**), *Psittacula derbiana* (**e**), *Neophena pulchella* (**f**) and *Barnardius zonarius* (**g**) species. Arrows and numbers correspond to location and size (in bp) of the DNA molecular marker run on the same gel.

The CR1 retroposon insertion in the CHD1i16 turned out to represent the Z/W polymorphism detected earlier by Fridolfsson and Ellegren [12] and named CHD1iA. The PCR primers used by Suh et al. [14] to amplify polymorphic fragments annealed to sequences internal relative to the CHD1iA primers, and therefore, the amplified products were smaller—553 bp instead of 593 bp for chromosome Z and 406 bp instead of 447 bp for chromosome W (as predicted based on the CHD1 sequence of *Gallus gallus*). Indeed, while tested in parallel to CHD1iA, the CHD1i16 marker gave almost identical patterns for seven parrot species (Figure 5) selected to represent most common variants of CHD1iA: 600 bp (Z) and 450 bp (W) bands (*Guaruba guarouba*—Figure 5a), 600 bp (Z) and 260 bp (W) bands (*Polytelis alexandrae*—Figure 5b, *Polytelis swainsonii*—Figure 5c), 1400 bp (Z) and 450 bp (W) bands (*Forpus crassirostris*—Figure 5d), 2500 bp (Z) and 450 bp (W) bands (*Psittacula derbiana*—Figure 5e), a single 600 bp (Z) band in male and female samples (*Neophena pulchella*—Figure 5f), 600 bp (Z) and very weak 450 bp (W) bands (*Barnardius zonarius*—Figure 5g). Consequently, the CHD1i16 marker was based on the same polymorphism as CHD1iA and therefore, was not further analyzed.

The LTR retroposon insertion in the Z chromosome located in the 10th intron of CHD1, named CHD1i9 following the original numbering [14], was expected to result in the larger product of the PCR amplification of the intron in the Z chromosome as compared to chromosome W. Indeed, in all analyzed parrots the Z band (if detected) was larger than the W band. Surprisingly, in *Gallus gallus*, the predicted size of the W band (5305 bp) was larger than the size of the Z band (1422 bp). 

**Figure 6:**
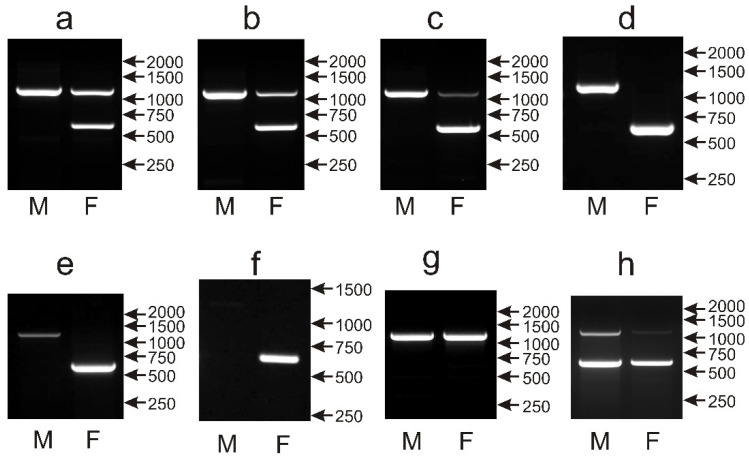
Different patterns of the PCR products representing the CHD1i9 marker obtained for male (M) and female (F) birds of *Loriculus galgulus* (**a**), *Platycercus icterotis* (**b**), *Psittacula eupatria* (**c**), *Polytelis alexandrae* (**d**), *Lorius chlorocercus* (**e**), *Trichoglossus haematodus* (**f**), *Amazona autumnalis* (**g**) and *Orthopsittaca manilata* (**h**) species. Arrows and numbers correspond to location and size (in bp) of the DNA molecular marker run on the same gel.

The potential utility of CHD1i9 as a sexing marker was evaluated by screening the set of 135 parrot species with the described PCR primers [14]. The typical pattern of two distinct PCR products in females (around 600 bp and 1150 bp) derived from the W and Z chromosomes respectively, and only the single 1150 bp product in males was observed in 96 of the screened species (Figure 6a–c). In 12 species, different patterns were observed, however sex determination was still possible: either the Z-chromosome band was detected in males, and only the W-chromosome band was present in females (Figure 6d,e) (*Bolborhynchus lineola*, *Polytelis alexandrae*, *Myiopsitta monachus*, *Chalcopsitta duivenbodei*, *Poicephalus meyeri*, *Lorius chlorocercus*, *Lorius garrulous*, *Lorius lory*, *Chalcopsitta atra*, *Trichoglossus euteles*), or the Z-chromosome band was not detected, and only the W-chromosome band was observed in females (Figure 6f) (*Probosciger aterrimus*, *Trichoglossus haematodus*). In 20 species, sex determination with the CHD1i9 marker was not possible because identical patterns were obtained after PCR in both female and male samples. Only the larger PCR product representing the Z chromosome was detected in males and females of almost all *Amazona* (*A. aestiva*, *A. albifrons*, *A. amazonica*, *A. auropalliata*, *A. autumnalis*, *A. barbadensis*, *A.* *brasilienis*, *A. collaria*, *A. dufresniana*, *A. festiva*, *A. finschi*, *A. guildingii*, *A. leucocephala*, *A. ochrocephala*, *A. oratrix*, *A. pretrei*, *A. tucumana*, *A. versicolor*, *A. vinacea*, *A. viridigenalis*), all *Pionus* (*P. chalcopterus*, *P. maximiliani*, *P. menstruus*, *P. senilis*), as well as *Alipiopsitta xanthops* and *Poicephalus gulielmi* (Figure 6g). The double bands corresponding to Z and W chromosomes were visualized in male and female *Orthopsittaca manilata* (Figure 6h).

Summarizing, the CHD1i9 marker, although not universal, can be successfully applied for the sexing of a large number of parrot species and, in several species (*Anodorhynchus hyacinthinus*, *Barnardius zonarius*, *Cacatua sulphurea*, *Neophema elegans*, *N. splendida*, *Platycercus caledonius*, *P. icterotis*), may act as a marker complementary to CHD1iA. 

**Figure 7:**
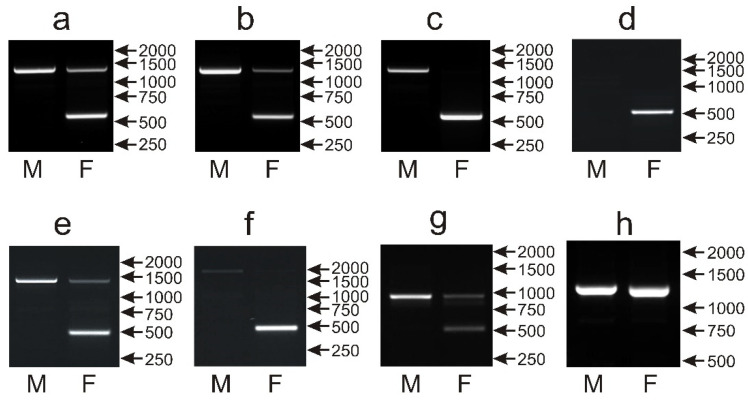
Different patterns of the PCR products representing the NIPBLi16 marker obtained for male (M) and female (F) birds of *Pyrrhura rupicola* (**a**), *Agapornis taranta* (**b**), *Agapornis canus* (**c**), *Chalcopsitta atra* (**d**), *Amazona albifrons* (**e**), *Psittacula cyanocephala* (**f)**, *Coracopsis vasa* (**g**) and *Psittacus erithacus* (**h**) species. Arrows and numbers correspond to location and size (in bp) of the DNA molecular marker run on the same gel.

The same set of 135 parrot species was applied to assess the performance of the NIPBLi16 amplicon as a molecular marker for sex determination based on the presence or absence of the CR1 retroposon insertion in the Z or W chromosomes, respectively. In 110 species, a typical pattern was obtained after the PCR with primers described by Suh et al. [14] containing the larger Z chromosome-derived band of about 1200 bp, present in both males and females, and the smaller W chromosome band of about 550 bp, observed only in females (Figure 7a,b). However, in 29 species, the intensity of the Z band was low in the female samples (Figure 7b). In the case of 10 other species (*Polytelis alexandrae*, *Trichoglossus haematodus*, *Alisterus scapularis*, *Chalcopsitta duivenbodei*, *Agapornis canus*, *Pyrrhura rhodocephala*, *Lorius chlorocercus*, *Trichoglossus euteles*, *Agapornis liliane*, *Psittacula alexandrii*), only the W band was visible (Figure 7c), but sex determination was still possible. Males and females could be also distinguished in eight other species wherein the W band had normal size and intensity in females, while the Z band was absent in males and females (*Chalcopsitta atra*—Figure 7d); the W band was smaller (below 500 bp), and the Z band was present in males and females (*Amazona agilis*, *Amazona albifrons*, *Amazona collaria*, *Amazona xantholora*, *Psittinus cyanurus*—Figure 7e); the W band was smaller, and the Z band was present only in males (*Psittacula cyanocephala*—Figure 7f), or, finally, the normal-size W band was visible, and the Z band had a smaller size (below 1000 bp) and was present in both male and female samples (*Coracopsis vasa*—Figure 7g). Sex determination with the NIPBL1i16 marker was not possible in seven species including four species from the *Poicephalus* genus (*Poicephalus gulielmi*, *Poicephalus senegalus*, *Poicephalus fuscicollis*, *Poicephalus meyeri*, *Psittacus erithacus*, *Myiopsitta monachus*, *Psilopsiagon aymara*), because only the Z band was detected in males and females, and the W band was invisible (Figure 7h).

Taking together, among all six markers analyzed in this study, the NIPBLi16 marker showed the widest spectrum, i.e., 128 out of 135 tested species could be successfully sexed. NIPBLi16 used in combination with CHD1iA ensures the sexing of all 135 species in our survey. 

### 3.7. Sexing with Only One Marker May Lead to Sex Misidentification—The Case of Genus Pyrrhura 

**Figure 8:**
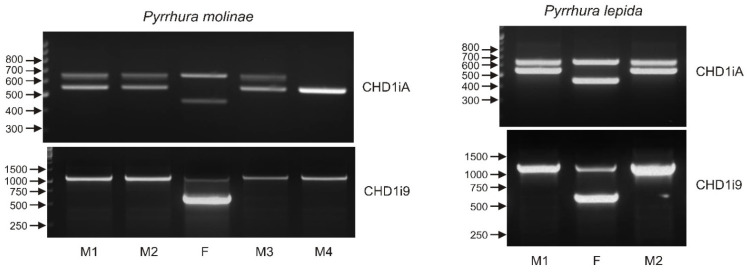
The patterns of the PCR products representing the CHD1iA (upper panel) and CHD1i9 (lower panel) markers obtained for male (M1, 2, 3, 4) and female (F) birds of *Pyrrhura molinae* and *Pyrrhura lepida*. Arrows and numbers correspond to location and size (in bp) of the DNA molecular marker run on the same gel.

Sex determination with molecular markers targeting gametologous genes on Z and W chromosomes is based on the assumption that, in males, the PCR products originating from two chromosomes Z have the same length and yield products visualized as a single band in electrophoresis. Therefore, two bands obtained after PCR are assumed to represent gene copies in chromosomes W and Z, and an individual presenting such pattern is considered as a female. Consequently, the PCR patterns obtained after sexing *Pyrrhura molinae* and *Pyrrhura lepida* nestlings with the CHD1iA marker (Figure 8) could be interpreted as the evidence of female sex of almost all individuals except M4. However, a closer inspection of the PCR product sizes suggested that this interpretation could be wrong. Only two of the female samples, marked as F in *P. molinae* and *P. lepida*, produced typical CHD1iA bands Z (650 bp) and W (450 bp) described in the literature [12] and most commonly observed in our survey of the Psittaciformes (Figure 3a, Table 1). For the remaining *Pyrrhura* nestlings presenting two CHD1iA bands, the Z fragment of 650 bp was visualized, while the typical W band was missing, and instead, a fragment of 550 bp was present. Moreover, the 550 bp band was obtained as the only PCR product in individual M4, indicating that M4 is male, and the band corresponds to the chromosome Z rearranged, probably by deletion within the CHD1iA amplicon resulting in the shorter amplification product. In conclusion, individuals M1, M2 and M3 of *P. molinae* and M1 and M2 of *P. lepida*, originally assumed to be females, were suspected to be males because they presented the typical CHD1iA band Z of 600 bp and the novel (rearranged) band Z of 550 bp. Indeed, sexing with the CHD1i9 marker confirmed this suspicion, and only individuals marked F in Figure 8 were identified as females. All *Pyrrhura* nestlings showed typical CHD1i9 patterns of male (single 1150 bp band) or female (600 bp and 1150 bp bands) individuals enabling unambiguous result interpretation. Taking together, the case of *Pyrrhura* nestlings exemplifies the misinterpretation risk associated with bird sexing when only one sex marker is used and indicates how important it is to apply multiple markers for sex identification.

### 3.8. Molecular Markers Detecting Polymorphisms of CHD1 and NIPBL Genes Can Be Amplified via Direct PCR without DNA Isolation from Blood

**Figure 9:**
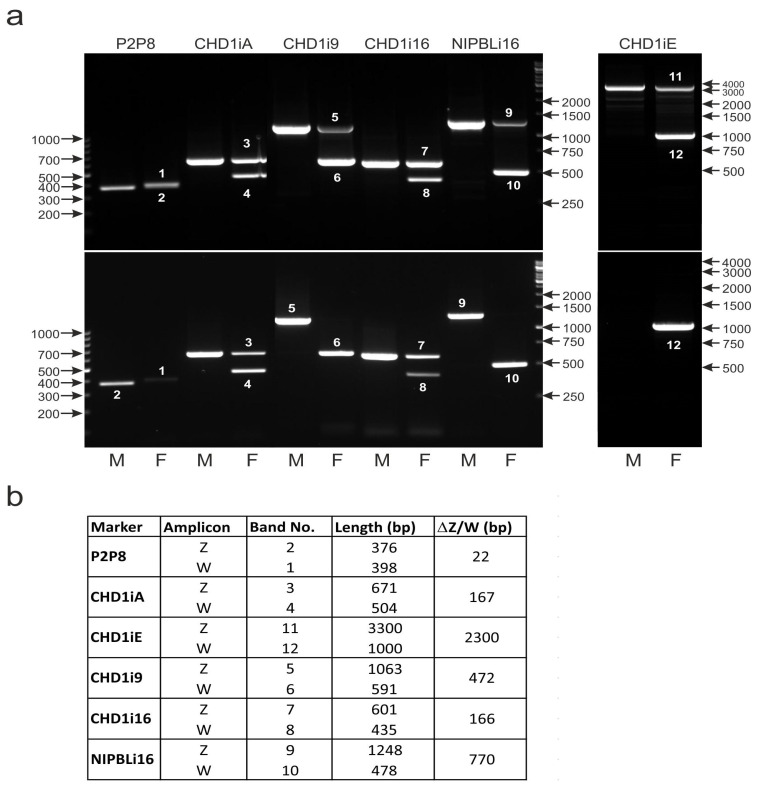
The P2P8, CHD1iA, CHD1iE, CHD1i9, CHD1i16 and NIPBLi16 markers. (**a**) The PCR products representing respective markers were obtained using as a template either DNA isolated from dried paper-blotted blood (upper panel) or DNA contained in dried paper-blotted blood placed directly in the PCR reaction mixture (direct PCR, lower panel). The samples of males (M) or females (F) of *Guaruba guarouba* were used. Arrows and numbers correspond to location and size (in bp) of the DNA molecular marker run on the same gel. (**b**) The table showing the length and the length difference of the PCR products corresponding to Z or W bands of each marker with the band numbers indicating their position in the electrophoresis gels in the upper and lower panels (**a**).

In direct PCR, the tissue sample is placed directly in the reaction mixture without prior template DNA isolation. The method is especially advantageous when numerous samples are analyzed since it allows the saving of time and decreases the costs of the procedure. Additionally, the contact of laboratory personnel with potentially biohazardous material is reduced as well as the risk of sample cross-contamination. Therefore, we checked whether the direct PCR approach could be applied in bird sexing with the described markers detecting Z/W chromosome polymorphisms of CHD1 and NIPBL genes. All markers allowed the distinguishing of male and female individuals of all analyzed Psittaciformes species when checked via direct PCR. As an example, we present the results of *Guaruba guarouba* couple sexing, obtained when template DNA was isolated from dried paper-blotted blood (Figure 9a—upper panel), or dried paper-blotted blood was directly (no DNA isolation) used for PCR (Figure 9a—lower panel). Both methods identified the sex of the analyzed individuals in the same way, but in the case of three markers, the Z band was not visible in the female samples (CHD1i9 and NIPBLi16) or in male and in female samples (CHD1iE) after direct PCR. The failure in the amplification of these amplicons was most probably caused by their relatively large size—more than 1000 bp in the case of CHD1i9 and NIPBLi16 and 3000 bp for CHD1iE. Direct PCR creates conditions wherein the amplification of longer molecules is more difficult due to contamination that is not removed during DNA isolation. The problematic amplification of large DNA fragments is especially pronounced when the template sequences located within the Z chromosome are present in only one copy per cell (in females). Taken together, these results indicate that direct PCR can be successfully used in sex determination in birds despite limitations in the amplification of large DNA fragments.

### 3.9. Strategy to Sex Birds with Combination of Markers

The CHD1iA was shown to correctly determine sex of many bird taxons including 83% of Psittaciformes tested in this study and many more mentioned in other reports [17,26,33]. Additionally, from a technical point-of-view, CHD1iA produces the best PCR pattern with easy to distinguish, relatively small but still well-separated Z and W bands that can be efficiently amplified in direct PCR. The CHD1i9 and NIPBLi16 polymorphisms between chromosomes W and Z were also confirmed as useful in sexing 80% and 95%, respectively, of the Psittaciformes species analyzed in this study. CHD1iE showed serious technical disadvantages, and consequently, we propose the use of this marker only for species for whom none or only one of the remaining markers works. Based on the results of our survey, we designed a strategy for sex determination using, at minimum, two and, at maximum, four markers to confirm the sex of the given bird, with at least two markers yielding conclusive and matching results. In the first step only two markers: CHD1iA and NIPBLi16 were used since they both give matching results in 72% of species wherein unambiguous PCR patterns were obtained (species marked with (1) in Table 1). Sex determination for such species was completed at this step. In the case of species wherein males and females are not distinguished by one of the markers (CHD1iA or NIPBLi16) or can be distinguished based on atypical patterns (shaded in Table 1) given by one or both of the above markers, the sexing procedure should be continued using the CHD1i9 marker. In our survey, this step was sufficient to sex an additional 27% of species (marked with (2) in Table 1) where conclusive results of sexing were obtained for at least two markers: CHD1i9, CHD1iA and/or NIPBLi16. Finally, in the species where only one of the three applied markers gave a conclusive result, the CHD1iE marker should be used (species (3) in Table 1). In our survey, we did not find the species where both CHD1iA and NIPBLi16 markers failed, and, almost always (except for *Orthopsittaca manilata* and *Poicephalus gulielmi*), at least two of the three CHD1iA, CHD1i9 and NIPBLi16 markers produced an informative pattern. Therefore, in Psittaciformes, the application of only three markers usually allows the confirmation of bird sex using at least two of them.

### 3.10. There Is No Universal Marker

Finding a universal molecular marker for the sex determination of a phylogenetically wide range of bird species has been an aim for many researchers in the last twenty-five years [12,18,27,29,41,42,43,44,45,46]. Particularly promising markers were length polymorphisms between introns of the genes having gametologous copies in the W and Z chromosomes. Several such polymorphisms were identified within the CHD1 gene, and some have been widely used in sex determination in birds. Despite numerous attempts, none of the tested markers appeared fully universal. Even markers shown to have very broad phylogenetic range failed to sex some species, although they were applied with success in other closely related species. Marker P2P8, known for its wide phylogenetic spectrum, was unsuccessful in sexing *Neophema pulchella* individuals, while it worked very well in sex determination in *Neopsephotus bourkii*—another species of the same *Pezoporini* tribe. Similarly, the marker did not distinguish male and female of *Cacatua sulphurea,* although it was successfully used for the sexing of *Nymphicus hollandicus*—both species belonging to the same *Cacatuidae* family. Moreover, very small size differences between the W and Z bands obtained after PCR with the P2 and P8 primers in the female sample make large-scale application of this marker technically challenging, at least in the Psittaciformes order. 

The CHD1iA marker gave one of the best results in our study, successfully sexing over 92% of the tested species and produced the chromosome W- and Z-specific PCR products of relatively small and well-distinguished sizes. However, even the CHD1iA marker was not universal and failed to sex nine species, including *Platycercus caledonius* and *Platycercus icterotis,* although the remaining three species of the *Platycercus* genus were sexed by the marker. The CHD1i9 marker sexed *Amazona agilis*, *Amazona* *farinosa*, *Amazona guatemalea*, *Amazona rhodocorytha* and *Amazona xantholora* but failed in the case of 20 other *Amazona* species. It also sexed *Poicephalus fuscicollis* but not *Poicefalus gulielmi*. The examples of CHD1iA and CHD1i9 markers indicate that inconsistencies in the application of molecular markers for avian sexing may occur even within the same genus. 

### 3.11. Sex Misidentification May Occur Due to Chromosomal Rearrangements

Recent studies of the evolution of avian sex chromosomes indicated that the W chromosomes of two species of the *Arini* tribe (*Myiopsitta monachus* and *Amazona aestiva*) had extremely diverse lengths due to divergent content and distribution of the repetitive sequences [47]. Polymorphism in chromosome Z in auklets described by Dawson et al. [48] resulted in two different chromosome Z copies present in one male. Such sex chromosome polymorphisms cause the risk of sex misidentification as we described for the *Pyrrhura* genus of the *Arini* tribe, wherein two bands originating from two Z chromosomes may be present in males but are normally interpreted as the female pattern. The chance of sex misidentification is especially high in massive surveys including species sexed for the first time, in particular when individuals of only one gender are analyzed. Therefore, sex determination in birds with only one molecular marker seems very risky.

### 3.12. Application of Multiple Markers Ensures Effective and Reliable Sexing 

In our studies the CHDi1A, CHDi9 and NIPBLi16 markers were found to be the most useful in sexing the analyzed Psittaciformes species. The markers produced easy-to-interpret patterns of relatively small and clearly distinct Z- versus W-specific PCR products. However, none of the best performing markers was able to distinguish between males and females of all the analyzed species. Moreover, patterns obtained for the given marker were not identical in all successfully sexed species, which further complicates Psittaciformes sexing. Accurate sexing may be additionally hampered by chromosomal rearrangements leading to sex misidentification. Therefore, based on our survey of Psittaciformes, we assume that reliable sexing requires conclusive and matching results of tests with at least two markers targeting different W/Z polymorphisms. In the case of some species, the application of the more challenging CHDi1E marker might be required to fulfill this condition. Our manuscript provides a detailed description of strategies and technical aspects of marker application and facilitates interpretation of the results presenting the whole range of patterns obtained for each marker in the wide spectrum of Psittaciformes species. Therefore, it can serve as a guide for those who would like to apply the sexing strategy we propose. 

## 4. Conclusions

The need for a robust sexing method for Psittaciformes is urgent, since parrots are both the most captivating and the most extinction-endangered bird order. The myth of the universal sexing marker has been debunked, because even the most promising markers failed to sex all species, and the application of only one marker was shown to cause a severe risk of sex misidentification. Therefore, we propose the use of a set of several markers (CHD1i16, NIPBLi16, CHD1i9 and CHD1iE) applied in a strategic order, optimized to minimize the work input (the number of necessary PCR reactions is reduced) and to ensure correct sex determination (the risk of sex misidentification is minimized) by at least two markers targeting independent Z/W polymorphisms. Furthermore, this approach greatly reduces experimental time and costs.

## Figures and Tables

**Table 1:** The Psittaciformes species analyzed in this study with the corresponding numbers and symbols of the PCR banding patterns obtained for the indicated markers and shown in Figures 1–9 and Figure S1.

SPECIES	Sexing Strategy ^b^	No	CHD1iE	CHD1iA	CHD1i9	NIPBLi16
*Agapornis canus* ^a^	2	68	―	3e	6b	7c
*Agapornis fischeri* ^a^	1	95	―	3a	6b	7a
*Agapornis liliane* ^a^	2	102	―	3a	6b	7c
*Agapornis nigrigenis* ^a^	2	62	4d	3e	6b	7a
*Agapornis personata* ^a^	1	73	―	3a	6c	7a
*Agapornis roseicollis*	1	24	4d	3a	6c	7a
*Agapornis taranta* ^a^	1	67	―	3a	6b	7a
*Alipiopsitta xanthops* ^a^	1	119	―	3a	6g	7a
*Alisterus scapularis* ^a^	2	39	4i	3d	6a	7c
*Amazona aestiva*	1	43	4a	3a	6g	7b
*Amazona albifrons*	1	78	―	3a	6g	7e
*Amazona agilis* ^a^	1	120	―	3a	6a	7e
*Amazona amazonica*	1	44	4a	3a	6g	7a
*Amazona auropalliata*	1	99	―	3a	6g	7a
*Amazona autumnalis*	1	79	―	3a	6g	7a
*Amazona barbadensis*	1	1	4a	3a	6g	7a
*Amazona brasiliensis*	1	121	―	3a	6g	7b
*Amazona collaria* ^a^	1	122	―	3a	6g	7e
*Amazona dufresniana* ^a^	1	123	―	3a	6g	7a
*Amazona farinosa*	1	94	―	3a	6c	7a
*Amazona festiva* ^a^	1	124	―	3a	6g	7a
*Amazona finschi*	1	59	4a	3a	6g	7b
*Amazona guatemalae* ^a^	1	125	―	3a	6c	7a
*Amazona guildingii* ^a^	1	132	―	3a	6g	7a
*Amazona leucocephala*	1	88	―	3a	6g	7b
*Amazona ochrocephala*	1	100	―	3a	6g	7a
*Amazona oratrix* ^a^	1	2	4a	3a	6g	7a
*Amazona pretrei*	1	76	―	3a	6g	7a
*Amazona rhodocorytha* ^a^	1	126	―	3a	6a	7a
*Amazona tucumana*	1	127	―	3a	6g	7a
*Amazona versicolor* ^a^	1	131	―	3a	6g	7a
*Amazona vinacea*	1	77	―	3a	6g	7a
*Amazona viridigenalis*	1	87	―	3a	6g	7b
*Amazona xantholora* ^a^	1	128	―	3a	6a	7e
*Anodorhynchus hyacinthinus*	2	53	4f	3h	6b	7a
*Aprosmictus erythropterus*	1	40	4h	3d	6c	7a
*Ara ararauna*	1	3	4a	3a	6b	7a
*Ara chloroptera*	1	52	4a	3a	6c	7b
*Ara glaucogularis*	1	54	4a	3a	6a	7a
*Ara macao*	1	42	4a	3a	6a	7a
*Ara militaris*	1	98	―	3a	6c	7a
*Ara rubrogenys*	1	4	4a	3a	6b	7a
*Ara severa*	1	109	―	3a	6a	7a
*Aratinga auricapillus* ^a^	1	108	―	3a	6b	7a
*Aratinga jandaya*	1	97	―	3a	6b	7a
*Aratinga nenday*	1	49	4f	3a	6b	7a
*Aratinga solstitialis*	1	26	4a	3a	6b	7a
*Aratinga weddellii* ^a^	1	117	―	3a	6c	7b
*Barnardius zonarius*	2	38	4c	3e	6b	7a
*Bolborhynchus lineola*	1	7	4h	3a	6e	7a
*Brotogeris jugularis* ^a^	1	112	―	3a	6c	7a
*Cacatua ducorpsii*	1	29	4a	3a	6a	7b
*Cacatua goffini*	1	51	4a	3a	6c	7b
*Cacatua moluccensis*	1	30	4a	3a	6b	7b
*Cacatua sulphurea*	1	8	4b	3a	6b	7a
*Calyptorhynchus banksii* ^a^	1	130	―	3a	6c	7a
*Chalcopsitta atra*	2	92	―	3a	6e	7d
*Chalcopsitta duivenbodei*	2	50	4h	3a	6e	7c
*Coracopsis vasa*	2	57	4a	3i	6b	7g
*Cyanoliseus patagonus*	1	9	4b	3a	6b	7a
*Cyanopsitta spixii* ^a^	1	135	―	3a	6b	7a
*Cyanoramphus novaezelandiae* ^a^	1	31	4a	3a	6a	7a
*Deroptyus accipitrinus*	1	80	―	3a	6b	7a
*Diopsittaca nobilis* ^a^	1	28	4a	3a	6b	7a
*Eclectus roratus*	2	58	4a	3f	6b	7b
*Enicognathus ferrugineus* ^a^	1	134	―	3a	6c	7b
*Enicognathus leptorhynchus* ^a^	1	133	―	3a	6c	7b
*Eolophus roseicapillus*	1	32	4c	3a	6b	7a
*Eupsittula aurea*	1	6	4a	3a	6b	7a
*Forpus coelestis* ^a^	1	106	―	3c	6a	7a
*Forpus crassirostris* ^a^	2	10	4i	3g	6a	7b
*Forpus xanthops* ^a^	1	96	―	3c	6a	7a
*Guaruba guarouba*	1	11	4c	3a	6b	7a
*Lathamus discolor* ^a^	1	104	―	3a	6b	7a
*Lophochroa leadbeateri* ^a^	1	55	4f	3a	6b	7b
*Loriculus galgulus*	1	60	4f	3a	6a	7a
*Lorius chlorocercus* ^a^	2	89	―	3a	6e	7c
*Lorius garrulus*	1	90	―	3a	6e	7b
*Lorius lory*	1	91	―	3a	6e	7b
*Melopsitacus undulatus*	1	25	4a	3a	6a	7b
*Myiopsitta monachus*	2	47	4a	3b	6e	7h
*Neophema elegans* ^a^	2	103	―	3h	6b	7a
*Neophema pulchella* ^a^	2	13	4a	3h	6b	7b
*Neophema splendida* ^a^	2	72	―	3h	6b	7a
*Neopsephotus bourkii*	1	12	4a	3a	6b	7a
*Nestor notabilis*	2	56	4j	3i	6b	7a
*Nymphicus hollandicus*	1	14	4a	3a	6b	7b
*Orthopsittaca manilata* ^a^	3	113	―	3i	6h	7b
*Pionites melanocephalus*	1	15	4c	3a	6b	7a
*Pionopsitta pileata*	1	101	―	3a	6b	7b
*Pionus chalcopterus*	1	69	―	3a	6g	7a
*Pionus maximiliani*	1	86	―	3a	6g	7b
*Pionus menstruus*	1	16	4a	3a	6g	7a
*Pionus senilis*	1	46	4a	3a	6g	7a
*Platycercus adscitus* ^a^	1	74	―	3a	6a	7a
*Platycercus caledonius* ^a^	2	110	―	3h	6a	7b
*Platycercus elegans*	2	17	4a	3e	6c	7a
*Platycercus eximius* ^a^	1	37	4a	3a	6b	7a
*Platycercus icterotis* ^a^	2	64	4a	3h	6a	7a
*Poicephalus fuscicollis* ^a^	2	116	―	3a	6b	7h
*Poicephalus gulielmi*	3	18	4a	3a	6g	7h
*Poicephalus meyeri*	2	118	―	3a	6e	7h
*Poicephalus senegalus*	2	33	4a	3a	6b	7h
*Polytelis alexandrae* ^a^	2	19	4h	3d	6d	7c
*Polytelis anthopeplus* ^a^	1	48	4h	3d	6b	7a
*Polytelis swainsonii*	1	20	4h	3d	6b	7b
*Primolius auricollis* ^a^	1	82	―	3a	6c	7a
*Primolius couloni* ^a^	1	61	4c	3a	6b	7a
*Primolius maracana* ^a^	1	41	4a	3a	6b	7a
*Probosciger aterrimus*	1	34	4a	3a	6f	7a
*Psephotus haematonotus* ^a^	1	21	4d	3a	6b	7a
*Psilopsiagon aymara* ^a^	2	71	―	3a	6b	7h
*Psittacara brevipes* ^a^	1	115	―	3a	6b	7a
*Psittacara finschi* ^a^	1	111	―	3a	6b	7b
*Psittacara mitrata* ^a^	1	85	―	3a	6b	7b
*Psittacara wagleri* ^a^	1	107	―	3a	6a	7a
*Psittacula alexandrii*	2	105	―	3f	6b	7c
*Psittacula cyanocephala*	2	70	―	3g	6c	7f
*Psittacula derbiana* ^a^	2	35	4e	3g	6c	7b
*Psittacula eupatria*	2	63	4g	3f	6b	7a
*Psittacula krameri*	2	45	4a	3f	6b	7a
*Psittacus erithacus*	2	22	4a	3a	6b	7h
*Psittinus cyanurus* ^a^	2	129	―	3g	6c	7e
*Purpureicephalus spurius* ^a^	1	81	―	3a	6b	7a
*Pyrhura roseifrons* ^a^	1	83	―	3a	6a	7a
*Pyrrhura frontalis*	1	27	4a	3a	6b	7a
*Pyrrhura molinae*	1	23	4a	3a	6b	7a
*Pyrrhura perlata*	1	75	―	3a	6a	7a
*Pyrrhura picta*	1	65	―	3a	6b	7a
*Pyrrhura rhodocephala*	2	84	―	3a	6b	7c
*Pyrrhura rupicola* ^a^	1	66	―	3a	6b	7a
*Rhynchopsitta terrisi* ^a^	2	114	―	3e	6b	7a
*Thectocercus acuticaudatus*	1	5	4a	3a	6b	7b
*Trichoglossus euteles*	2	93	―	3a	6e	7c
*Trichoglossus haematodus*	2	36	4h	3a	6f	7c

^a^ species sexed for the first time in these studies. ^b^ Sexing strategy: (1)—two markers: CHD1iA and NIPBLi16 are used; (2)—CHD1i9 marker is used together with CHD1iA and/or NIPBLi16; (3)—CHD1iE marker is used together with one of the following markers: CHD1iA, CHD1i9, NIPBLi16. Grey and dark-gray shades correspond to atypical PCR patterns that allow and do not allow distinguishing bird sex, respectively.

**Table 2:** Phylogenetic distribution within the Psittaciformes order of genera (in bold) represented by at least one species analyzed in this study.

Superfamily	Family	Subfamily	Tribe	Genus
Strigopoidea	Strigopidae			*Strigops*
	Nestoridae			***Nestor***
Cacatuoidea	Cacatuidae	Nymphicinae		***Nymphicus***
		Calyptorhynchinae		***Calyptorhynchus***
		Cacatuinae	Microglossini	***Probosciger***
			Cacatuini	***Cacatua**, Callocephalon, **Eolophus**,* ***Lophochroa***
Psittacoidea	Psittacidae	Psittacinae		***Poicephalus, Psittacus***
		Arinae	Arini	***Anodorhynchus, Ara, Aratinga,*** ***Cyanoliseus, Cyanopsitta, Deroptyus,***
				***Diopsittaca, Enicognathus, Eupsittula***, ***Guaruba**, Leptosittaca, Ognorhynchus,*
				***Orthopsittaca, Pionites, Primolius,*** ***Psittacara, Pyrrhura, Rhynchopsitta***, ***Thectocercus***
			Androglossini	***Alipiopsitta, Amazona, Brotogeris,*** *Graydidascalus, Hapalopsittaca,*
				***Myiopsitta, Pionopsitta, Pionus**, Pyrilia,* *Triclaria*
			Amoropsittacini	***Bolborhynchus**, Nannopsittaca,* ***Psilopsiagon**, Touit*
			Forpini	***Forpus***
	Psittrichasidae	Psittrichasinae		*Psittrichas*
		Coracopseinae		***Coracopsis***
	Psittaculidae	Platycercinae	Platycercini	***Barnardius, Cyanoramphus**, Eunymphicus,* ***Lathamus**, Northiella,*
				***Platycercus**, Prosopeia, **Psephotus**,* ***Purpureicephalus***
			Pezoporini	***Neophema, Neopsephotus**, Pezoporus*
		Psittacellinae		*Psittacella*
		Loriinae	Loriini	***Chalcopsitta**, Charmosyna, Eos,* *Glossopsitta, **Lorius**, Neopsittacus,*
				*Oreopsittacus, Phigys, Pseudeos,* *Psitteuteles, **Trichoglossus**, Vini*
			Melopsittacini	***Melopsittacus***
			Cyclopsittini	*Cyclopsitta, Psittaculirostris*
		Agapornithinae		***Agapornis**, Bolbopsittacus, **Loriculus***
		Psittaculinae	Polytelini	***Alisterus, Aprosmictus, Polytelis***
			Psittaculini	***Eclectus**, Geoffroyus, Prioniturus,* ***Psittacula, Psittinus**, Tanygnathus*
			Micropsittini	*Micropsitta*

## Data Availability

Our research does not involve any nucleotide or amino acid sequence data that should be archived in publicly accessible repositories. All PCR patterns obtained are presented in figures included in the manuscript.

## Supplementary Materials

The following are available online at https://www.mdpi.com/article/10.3390/genes12060878/s1, Figure S1: The PCR products obtained during optimization of the amplification conditions for the CHD1iE marker using DNA isolated from the blood of male (M) or female (F) *Guaruba guarouba* as the template. (a) Results obtained when the original Program 1 was applied [23]. (b) Results obtained when Programs 2–5 were tested with annealing temperatures varying in the range of 42–52 °C). Arrows and numbers correspond to location and size (in bp) of the DNA molecular marker run on the same gel.
