# Supplementary material for: New Bird Sexing Strategy Developed in the Order Psittaciformes Involves Multiple Markers to Avoid Sex Misidentification: Debunked Myth of the Universal DNA Marker
Source: Genes (Basel). 2021 Jun 7;12(6):878. doi: 10.3390/genes12060878 (PMC8230142; doi:10.3390/genes12060878)
Supplement: Supplementary file 1 [file genes-12-00878-s001.zip › genes-1240096-supplementary.pdf]

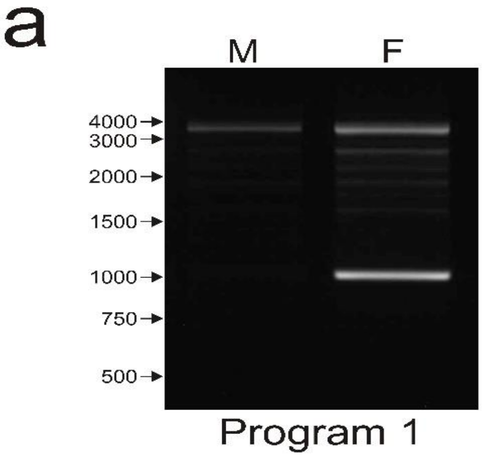

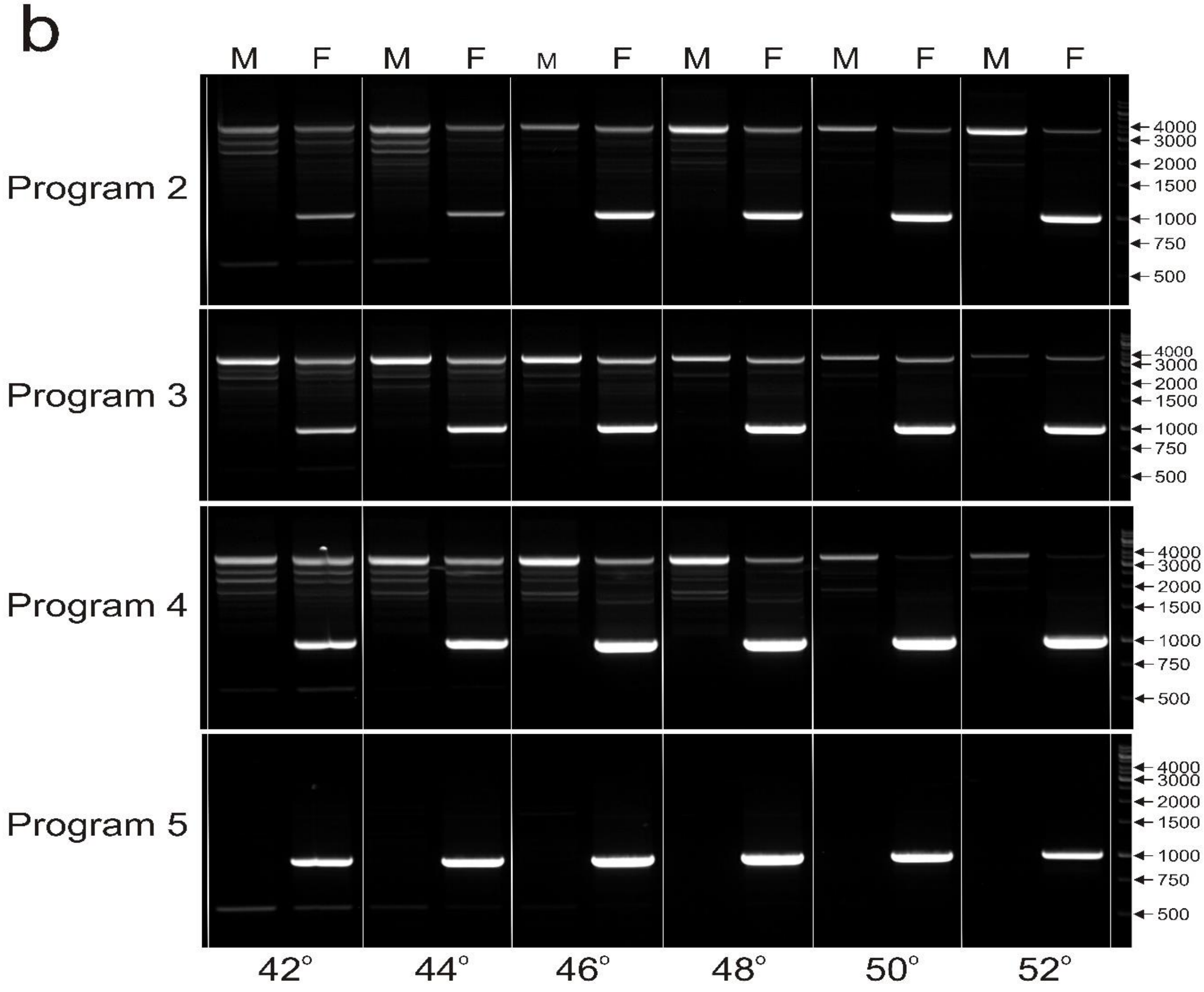

Figure S1. The PCR products obtained during optimization of the amplification conditions for the CHD1iE marker using DNA isolated from blood of male (M) or female (F) *Guaruba guarouba* as the template. (a) Results obtained when the original Program 1 was applied [23]. (b) Results obtained when Programs 2 – 5 were tested with annealing temperatures varying in the range of 42ºC - 52ºC). Arrows and numbers correspond to location and size (in bp) of the DNA molecular marker run on the same gel.
